# Synergies of Extracellular Vesicles and Microchimerism in Promoting Immunotolerance During Pregnancy

**DOI:** 10.3389/fimmu.2022.837281

**Published:** 2022-07-01

**Authors:** José M. Murrieta-Coxca, Paulina Fuentes-Zacarias, Stephanie Ospina-Prieto, Udo R. Markert, Diana M. Morales-Prieto

**Affiliations:** Placenta Lab, Department of Obstetrics, Jena University Hospital, Jena, Germany

**Keywords:** cross-dressing, immunotolerance, microchimerism, pregnancy, extracellular vesicles (EV), allorecognition

## Abstract

The concept of biological identity has been traditionally a central issue in immunology. The assumption that entities foreign to a specific organism should be rejected by its immune system, while self-entities do not trigger an immune response is challenged by the expanded immunotolerance observed in pregnancy. To explain this “immunological paradox”, as it was first called by Sir Peter Medawar, several mechanisms have been described in the last decades. Among them, the intentional transfer and retention of small amounts of cells between a mother and her child have gained back attention. These microchimeric cells contribute to expanding allotolerance in both organisms and enhancing genetic fitness, but they could also provoke aberrant alloimmune activation. Understanding the mechanisms used by microchimeric cells to exert their function in pregnancy has proven to be challenging as per definition they are extremely rare. Profiting from studies in the field of transplantation and cancer research, a synergistic effect of microchimerism and cellular communication based on the secretion of extracellular vesicles (EVs) has begun to be unveiled. EVs are already known to play a pivotal role in feto-maternal tolerance by transferring cargo from fetal to maternal immune cells to reshape their function. A further aspect of EVs is their function in antigen presentation either directly or on the surface of recipient cells. Here, we review the current understanding of microchimerism in the feto-maternal tolerance during human pregnancy and the potential role of EVs in mediating the allorecognition and tropism of microchimeric cells.

## Introduction

The Nobel Prize Laureate Sir Peter Medawar has become known for the formulation of the so-called immunological paradox of pregnancy. He found it surprising that, despite expressing foreign paternal antigens, the fetus remains in the mother’s uterus instead of being rejected in the way that a skin graft of paternal tissue would be (1, 2). He proposed three mechanisms by which the fetus could avoid recognition by the maternal immune system: the anatomical separation between mother and fetus by the placenta, the immaturity of fetal antigens which impairs their ability to elicit a maternal immune response, and the immunological inertness of the maternal immune system during pregnancy (3, 4). Medawar’s work has set the first milestone in the study of reproductive immunology, but despite his efforts and those of several medical scientists throughout the last decades, the mechanisms that govern feto-maternal tolerance are still only partly understood.

During normal human pregnancy, a natural bidirectional immune regulation allows an intimate contact between maternal and fetal cells at the maternal-fetal interface in a way that a state of immune non-reactivity specific to particular antigens is established. Thereby, the developing immune system of the fetus tolerates protein products derived from polymorphic genes that are expressed by the mother but are not inherited, so-called non-inherited maternal antigens (NIMAs), while the maternal immune system tolerates the inherited paternal antigens (IPAs) expressed by the fetus (5, 6).

Although the placenta represents an important physical barrier between mother and fetus, it is permeable to a multitude of signal substances and cellular types which also contribute to the establishment of immune tolerance. Small quantities of both, mature and progenitor cells can cross the placenta in a two-way cell trafficking. These cells are known as microchimeric cells and play a key role in the regulation of alloresponses towards NIMAs and IPAs (5). Additionally, the communication between placental cells and maternal immune cells mediated by extracellular vesicles (EVs) is one of the mechanisms involved in allotolerance that is a current research focus. EVs secreted by fetal tissues have the potential to transfer fetal proteins and nucleic acids to maternal cells reshaping their function. Here, we review the current understanding of microchimerism in the feto-maternal tolerance during human pregnancy and the role of EVs in mediating alloresponses, cell trafficking, and tropism of microchimeric cells.

## History of Microchimerism

Back in 1945, Ray Owen reported the presence of two distinct blood groups in fraternal twin cows: their own and that of their twin (7). To explain this, he proposed a cell interchange between the bovine twin embryonal cells *in utero*, ancestral to the erythrocytes of the adult animal. The exchanged cells could become established in the hematopoietic tissues of their co-twin hosts, providing a source of blood cells distinct from those of the host, presumably throughout its life, which is the conceptual foundation of acquired immunological tolerance (7). A second publication of the same group years thereafter stated that the actively acquired Rh tolerance to Rh antigens displayed by Rh-negative women was related to their Rh-positive mothers. The possible explanation was the prenatal exposure to Rh antigens or Rh-positive cells derived from the mother (8).

Owen´s research was picked up by Burnet and Fenner as evidence of a phenomenon they named “tolerance”, placing it in the context of the “self or non-self” hypothesis whereby an organism recognizes “self” and actively defends against pathogens and tissues that are non-self (9, 10). This hypothesis was also tested by Sir Medawar and Billingham by inoculating fetuses of one mouse strain with cells from a second and later in mice adulthood, performing skin homografts from the original donor strain. These grafts were accepted, but those from a third, unrelated mouse strain, were rejected, agreeing with the fact that “self” is defined during embryonic development as Burnet and Owen had hypothesized (10, 11). Contemporarily, the British physician Dunsford found two separate blood types in the blood of a donor who had a twin brother that had died young; a phenomenon he called “blood group chimera” (12, 13). These studies helped lay the groundwork for understanding the process by which two different cell populations can be found in an individual. This became a fundamental discovery in immunology, providing new ideas for organ transplantation but it also resulted fundamental in understanding human pregnancy as actively acquired tolerance that may occur naturally by the incorporation of maternal cells into a fetus during normal development (11). These studies also set the definition of chimera as an organism whose cells derive from two or more distinct zygote lineages (14), which continues to be the meaning of the term at present times.

## Pregnancy-Derived Microchimerism

Currently, microchimerism is understood as the presence of less than 1% allogeneic cells or DNA housed by an individual or an organ (5, 15). Tissue microchimerism is considered to be primarily pregnancy-derived, as a bidirectional cross-placental cell trafficking occurs between fetus and mother. The decidua-trophoblast interface plays a permissive role allowing stem cells and leukocytes, among others, to be transferred from maternal tissues to fetal tissues giving rise to maternal microchimerism, and from fetal tissues to maternal tissues generating fetal microchimerism (5, 15–18). Cell-free fetal DNA can be detected in maternal blood from the fourth week of gestation (19, 20), increasing in concentration as pregnancy progresses, with a sharp increase over the last 8 weeks of pregnancy (21), but becoming undetectable at day 1 after delivery, implying that its presence is limited to the current pregnancy (22). Conversely, pregnancy-derived microchimerism has proven to be long-lasting as a small number of maternal cells persist in her offspring until adulthood, and fetal cells can be found in the mother decades after parturition (23–29). Further, microchimerism is asymmetrical, as more fetal cells are transferred to the mother - than maternal cells to the fetus (15, 16, 30, 31). Yet, the microchimeric cells seem to be more decisive for the development of the fetus than for the mother possibly due to its nascent immune system (32).

The transfer of fetal material into the maternal system was first identified in 1893 as the presence of multinucleated cells with characteristic morphology of placental cells in diverse tissues of pregnant women who died from eclampsia (33). Nowadays, it is proven that the number of fetal microchimeric cells rises in the maternal body according to the gestational age. They consist of numerous cell types such as trophoblast cells, lymphocytes, hepatocytes, erythroblasts, and mesenchymal progenitor stem cells (30). In humans, fetal microchimeric cells infiltrate maternal tissues as early as seven weeks of gestation, differentiate, replicate and proliferate therein (19). In mice, fetal microchimeric cells appear detectable in the mother in the second week of pregnancy (34–36). Remarkably, the distribution of fetal microchimeric cells in the organs of pregnant women is not homogeneous, with the lung being the most chimeric organ followed by the spleen, liver, kidney, brain, and heart (37). Combining results from humans describing the specific localization of fetal cardiomyocytes in the maternal heart (24) and from mice locating fetal cells in maternal lungs, spleen, heart, liver, kidney, and brain (36), suggest the existence of specific mechanisms driving fetal cell tropism.

Likewise, maternal cells expressing surface markers of T and B lymphocytes, and leukocytes, leukocytes have been found in fetal tissues from the second trimester of pregnancy (38), and in immunocompetent adult offspring (25). Yet, some questions are still raised related to their involvement in the context of tolerance induction, inflammatory and autoimmune disorders in the offspring (36, 38–41).

During pregnancy, maternal microchimerism induces fetal allotolerance towards NIMAs, whereas fetal microchimerism is associated with maternal tolerance towards IPAs (36, 39–41). These effects could become long-lasting on the fetal immune system, while they seem to be only temporary on the maternal side (42, 43). The majority of NIMAs and IPAs are encoded by polymorphic genes belonging to the major histocompatibility complex (MHC) class I and II (5, 44). These molecules define the degree of maternal-fetal mismatch and the immune response against non–self tissues or allogeneic products.

In a normal immune scenario, microchimeric cells can be recognized and eliminated by the host immune system, but during pregnancy, a set of immunological adaptations is triggered to guarantee allotolerance both locally and systemically (4, 29, 45). On cellular level, the generation of Treg cells plays a fundamental role in development of immune tolerance. It occurs upon stimulation of thymus-derived naive CD4^+^CD44^low^ cells with cell-extrinsic stimuli including pregnancy-related hormones, such as progesterone and glucocortocoids, Transforming growth factor β - TGF-β TGF-β, and semiallogeneic fetal antigens (46). A potential mechanism of immune tolerance driven by fetal antigen-specific Treg cells is the suppression of decidual inflammation (46). It has been proposed that these cells could persist in mother tissues as "memory cells" and can expand in subsequent pregnancies enforcing fetal immune tolerance (46).

Also CD8+ regulatory or suppressor T cells may contribute to fetal tolerance [summarized in (46)], which is more pronounced in later pregnancy (47, 48). In mice, proliferation of CD8+ T cells increases at midgestation in mice, simultaneously with increased detection of systemic fetal antigens (49). CD8+ T regulatory or suppressor cells may reduce antibody production in B cells (50).

Additionally, alike malignant, microchimeric cells have developed strategies to invade normal tissue including blood vessels and to avoid destruction by the host immune system by acquiring surveillance and adaptive properties for immune evasion (4). This is partly caused by unique, specific subsets of HLA molecules on trophoblast cells: syncytiotrophoblast (STB) and villous cytotrophoblast lack all MHC Class I and MHC Class II molecules, so that T cells cannot bind to the main placental interface. Although invasive extravillous trophoblast cells (EVT) do express HLA class I, they lack HLA-A and HLA-B antigens. Instead, they express the non-classical HLA-E, HLA-F, and HLA-G. Likewise, EVT lack MHC Class II antigens so they cannot act as antigen presenting cells (APC) initiating direct allo-recognition by CD4^+^ T helper cells (4, 51). This non-classical pattern of HLA expression partly explains the maternal immunotolerance to paternal HLA molecules in the fetus (4).

Additional mechanisms to avoid maternal rejection include the expression of HLA-G receptor ILT2 by decidual T cells, which after recognition of fetal expressing HLA-G cells, drives a tolerogenic response. HLA-G also inhibits cytolytic T cell functions (52), and some isoforms of HLA-G could be transported from trophoblast to maternal immune cells *via* EVs, which can induce immune tolerance (53) or alter immune cell proliferation. Likewise, *via* specific ligands dNK cells are also modulated by non-classical HLA patterns on EVT, which switch their cytotoxic function to a tolerogenic behavior (4, 54, 55). The mechanisms of maternal tolerance induction to other microchimeric cells than trophoblast cells are still unknown.

It has been proven that the exposure of the fetal immune system to NIMAs by placental cell trafficking gives rise to an early NIMA-specific regulation through the induction and maintenance of allospecific CD4^+^CD25^+^ regulatory and CD8^+^ T cells during fetal life (5, 56, 57). These cells produce TGF-β an immune regulatory cytokine that suppresses the anti-NIMA response of fetal T effector cells, mitigating maternal–fetal conflict to enforce tolerance (7, 56, 57). Other functional roles that may be partially attributable to pregnancy-derived microchimerism include the amelioration of autoimmune disorders in women with a higher number of prior pregnancies (46, 58, 59 **)**, the replacement of injured human and murine maternal cells by fetal microchimeric cells that migrate to the site of damage and proliferate locally (60–65), and the potential adaptation of the maternal breast physiology and induction of milk supply suggested by the presence of fetal microchimeric cells (66, 67). Likewise, maternal cells may support fetal immune cell development, these potential protective effects of maternal microchimeric have been evidenced in mice by the presence of maternal cells in primary and secondary lymphoid organs before the fetal immune system is fully developed in healthy and immune-deficient offspring (29, 56). In humans this has been suggested by the presence of expanded populations of circulating maternal T cells which improve the health of offspring by augmenting host defense against microorganisms (68). Nevertheless, as the number of microchimeric cells is very low per definition, factors secreted by these cells should play a pivotal role in their function.

## Historical Milestones of Research on EVs in Pregnancy

The beginning of the field of EV biology could be dated to the early research on blood coagulation, with the discovery of Chargaff & West in the 1940s of a “particulate fraction” that sedimented at high speed and contained breakdown products of blood corpuscles with clotting potential (23, 69). It was only until the report of pioneering experiments with platelets that the presence and structure of those cell-free particles and their biological relevance began to be described. In 1967, Peter Wolf published electron microscopy images of a particle-like material originating from platelets, which could be high-speed sedimented and was distinguishable from intact platelets. These particles named “platelet dust” are currently known as EVs (70). Later in 1971, Neville Crawford published further images of EVs, this time being named “microparticles”, described partially their cargo, and suggested that EVs originated either in the surface membrane or within the intracellular membrane structures (71). Over the following decade and with the parallel improvement of analytic techniques, the biogenesis of different populations of EVs was described (72, 73) and the nomenclature of EVs including the term exosomes started to be coined (74, 75).

Summarizing the study of EVs in the context of pregnancy is also challenging due to the heterogeneous nomenclature used in the first studies. It can be argued that it dates back to 1974, when a simple procedure was proposed to obtain “membrane-bound bodies” from human placental villous by mechanical disruption (76). This method with minor modification is still in use for the isolation of EVs from placenta explants. Following that discovery, several groups reported both stimulatory and suppressor immune responses upon the treatment of mixed lymphocyte cultures with these preparations, which suggested their role in the regulation of immune responses and maternal allogeneic recognition during pregnancy (77, 78). Probably, one of the breakthrough events in the field was the finding of placental EVs in the maternal peripheral plasma, and the elevated levels in women suffering of Preeclampsia (PE) (79). This suggested a role of EVs in the systemic alterations for immune tolerance during pregnancy but also opened the field to the use of EVs as biomarkers for pregnancy pathologies.

In 2005 the first EV meeting took place, and since then there are regular meetings of the formed International Society for Extracellular Vesicles (ISEV) housing thousands of participants working in the study of EVs in different contexts including pregnancy. The ISEV endorses the description of EV as the generic term for particles naturally released from cells, which are delimited by a lipid bilayer, do not contain functional nucleus and cannot replicate (80). Cumulative evidence has demonstrated that EVs contribute to the transfer of functional elements, which constitutes a pivotal mechanism of cell-cell communication (81). EVs carry functional molecules including lipids, proteins, RNA (mRNA, miRNA, lncRNA), as well as DNA molecules (frequently related to pathological states) (81–84). EVs are released in an evolutionarily conserved manner by cells ranging from organisms such as prokaryotes to higher eukaryotes and plants. Based on their physical characteristics such as size and density, or specific markers of the intracellular origin, different EV subtypes can be defined (80). Depending on their biogenesis, three main EV populations have been described: Apoptotic bodies, which are released by plasma membrane blebbing occurring during apoptosis; microvesicles (also known as microparticles or ectosomes), which include vesicles of different sizes that are released from the plasma membrane; and exosomes, which are generated from intraluminal vesicles (ILV) by invagination of the endosomal membrane and accumulate in multivesicular bodies (MVB). Upon fusion of MVB with the plasma membrane, exosomes are released into the extracellular environment (85–87). This classification often overlaps with that by size because small (sEVs; <200nm) and medium/large EVs (m/lEVs; >200nm) are in most cases enriched fractions of exosomes and microvesicles, respectively (80). To identify and isolate trophoblast-derived EVs, specific placenta markers such as placental alkaline phosphatase (PLAP) (88), syncitin-1/2 (89), and HLA-G (90) are used. Likewise, tetraspanin CD63, ALIX, and TSG101 are considered general markers for exosomes as they localize predominantly to late endosomes and play an important role in sorting ILV (91, 92). Nevertheless, there is still a lack of appropriate and ubiquitous markers to differentiate other EV types and trace their cellular origin.

## EVs as Mediators of Immunological Adaptations During Pregnancy

The human placenta releases rising concentrations of EVs during pregnancy, which are distributed to other organs and cells (93). The major producer of placental EVs is the STB covering the maternal villi and directly in contact with the maternal bloodstream (79). The STB also releases a particular population of large vesicles denominated syncytial nuclear aggregates (SNAs) containing fetal DNA, RNA, and organelles (94, 95). A cross-talk between the placenta and the maternal immune system is established *via* EVs, as placenta-derived EVs are incorporated by neighboring and distant maternal immune cells (96–99), while EVs produced by maternal immune cells modulate placental responses (100, 101).

**Figure 1:**
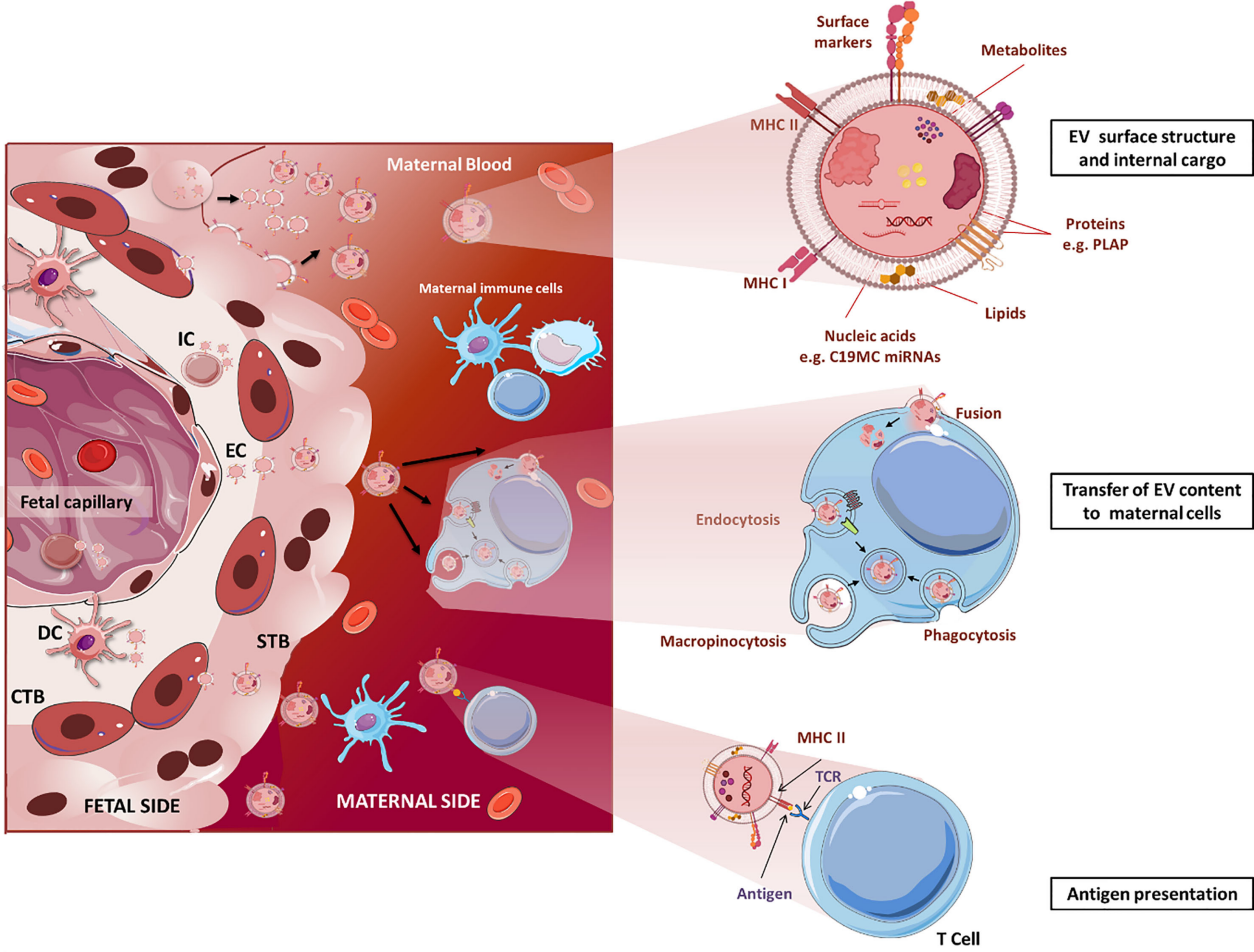
Structure and immunological functions of fetal EVs in pregnancy. Fetal cells, including endothelial, immune, dendritic, and CTB cells, and especially STB, release great amounts of EVs to the maternal circulation. Upper: EV surface and internal cargo. Middle: Fetal EVs transfer their cargo to maternal immune cells by different mechanisms including endocytosis, phagocytosis and membrane fusion. Lower: EVs can act as APCs directly interacting with maternal T cells and trigger immunotolerance or activation. STB, syncytiotrophoblast; CTB, cytotrophoblast; IC, immune cell; EC, endothelial cells; DC, dendritic cells. For the sake of clear illustration, the size of structures is not displayed in realistic proportions. The figure was drawn using pictures from 105 (http://smart.servier.com/).

Studies on placental EVs have characterized their surface and internal cargo revealing the presence of immunomodulatory factors [e.g. Fas ligand, TRAIL (102)], minor histocompatibility antigens [e.g. RPS4 (103)], glycoproteins [e.g. syncytin-1 (89)] and miRNAs (104), among others. Transfer of these signals from fetal tissues to maternal T cell, NK cell and macrophages *via* EVs may influence maternal immune responses and induce epigenetic reprogramming (**Figure 1**). Uptake of placental EVs *in vitro* has been demonstrated to occur in nearly every tested cell, and in most cases, it has been associated to measurable effects, however this can be different when other cell types and signals are also present as it is the situation *in vivo* (106–109). Recent studies have suggested that the EV uptake ratio could be cell-dependent with some cells such as macrophages and mature dendritic cells incorporating more EVs than monocytes and immature dendritic cells (110). However, the literature is heterogeneous when comparing other cell populations: a study reported T lymphocytes incorporating more placental-lEVs than B and NK cells (90), while in a second study, uptake of trophoblast-derived lEVs and sEVs is higher in NK compared to T cells (107). Standardization of the sample collection, isolation, and characterization protocols will allow comparisons between studies to clarify the specificity and mechanisms of placental- and trophoblast-EV uptake by immune cells.

The function of placental EVs remains largely unclear but most of the studies report their involvement in immune responses related to induction of immune tolerance by different mechanisms. Most of the immunoregulatory surface molecules of the STB have been detected on its EVs where they are supposed to fulfil similar functions. In the following we list several examples that have been investiagted. Trophoblast-derived EVs containing FasL might eliminate activated maternal T cells (102, 111). Human placenta explants release exosomes bearing ligands (MHC class I chain-related (MIC) proteins A and B, UL-16 binding proteins) of the NKG2D receptor on NK cells, cytotoxic T cells and γδ T cells. Binding of the ligands to NKG2D leads to reduced *in vitro* cytotoxicity without affecting the perforin-mediated lytic pathway (97). Furthermore, placental exosomes suppress in T cells the expression of CD3-zeta chain and Janus kinase 3 (JAK3) but induce suppressor of cytokine signalling 2 (SOCS2) which may favor the expansion of lymphocytes with suppressive phenotypes, such as Treg cells (112).

Previously, we have shown that EVs enriched from choriocarcinoma cell lines transfected with a specific miRNA carry large amounts of this miRNA. Upon co-incubation with T and NK cells *in vitro*, the presence of specific miRNAs is able to influence their proliferation. This suggests a specific role of EVs depending on their specific cargo (107, 113). Specific features of EVs seem to be also involved in pathological conditions. A recent study reported a different effect for STB-derived small and large EVs, which upregulate pro-inflammatory cytokines in THP-1 macrophages when isolated from normal pregnancy and further increase significantly when isolated from serum of patients with PE (114). More specific effects of placental EVs on immune cell populations have been reviewed in detail previously (55, 98, 114, 115) and will not be further addressed here.

An additional aspect of EVs in feto-maternal tolerance is their function in the antigen presentation (**Figure 1**). This can occur either directly *via* MHC-peptide complexes on the EV surface, or indirectly *via* MHC cross-dressing of antigen (Ag)-presenting cells (APCs), a process that refers to the acquisition of intact MHC molecules pre-loaded with antigen (Ag)-derived peptides by leukocytes, in particular APCs (116). These mechanisms are of interest in the establishment and maintenance of pregnancy-derived microchimerism and will be described in detail in the next section.

## Conceivable Mechanisms of Microchimerism-Induced Allorecognition and Immunotolerance Involving EVs

**Figure 2:**
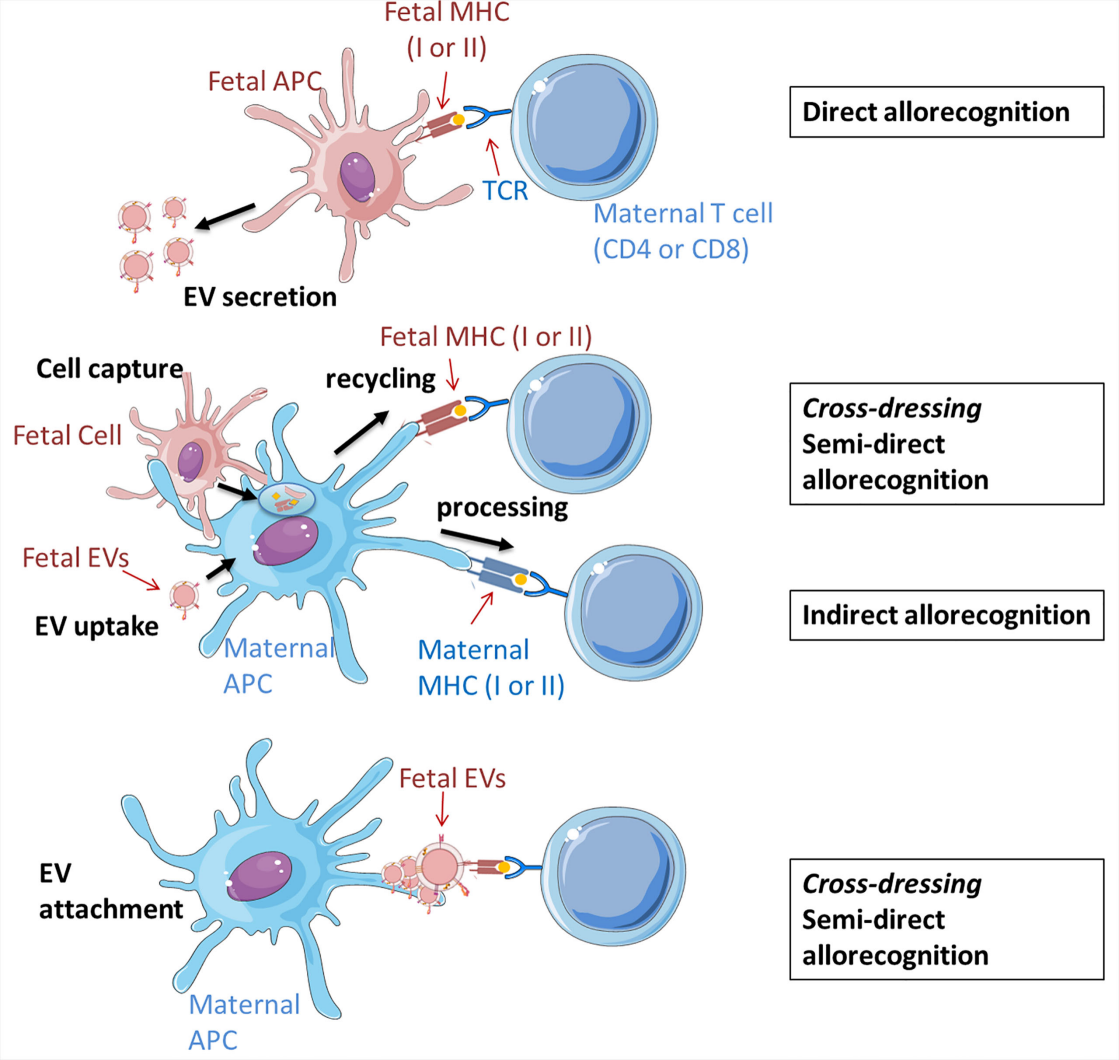
Potential scenarios of EV involvement in the fetal allorecognition by maternal T cells. Maternal cells are shown in blue and fetal cells and EVs are shown in red. Up: Direct pathway. Fetal APCs, which secrete EVs to the intercellular space, present peptides (auto- or alloantigens) *via* MHC (I or II) molecules to immunocompetent maternal T cells. Middle: In the semi-direct pathway, fetal allogeneic MHC molecules (I or II) are acquired *via* capture and uptake of fetal cells (entire or only their cell membrane) or their secreted EVs, and then recycled and expressed on the maternal APC surface. APC can present antigens bound to fetal MHC molecules to maternal T cell receptors. The indirect pathway implies the processing of fetal proteins from EVs or cells and the presentation of deriving peptides by maternal MHC II complexes to T maternal cells. Down: Fetal-derived EVs cross-decorate maternal APCs with intact MHC-peptide complexes that can be recognized by T cells similar to the semi-direct pathway. All displayed processes involve co-stimulatory interactions between APCs and T cells which are decisive for subsequent reactions, which may be tolerogenic, immunoregulatory or –stimulatory. The figure was drawn using pictures from 105 (http://smart.servier.com/) and was based on previous revisions (5, 116).

Classically, there are two distinct and non-exclusive mechanisms used by host immune cells to recognize alloantigens: the indirect and direct pathways (**Figure 2**). In the direct pathway, antigen presenting cells (APC) from the graft (in the context of pregnancy the embryo/fetus) present alloantigen complexes *via* MHC Class I and Class II that are recognized respectively by CD8 and CD4 T cells CD8^+^ and CD4^+^ T cells of the host (the mother). In the indirect pathway, graft alloantigens (typically MHC antigens) are internalized by host APC (mostly dendritic cells), processed, and presented as peptide fragments associated to host MHC molecules for recognition by its T cells (117). Direct and indirect allorecognition pathways are poorly studied in pregnancy, being a unique situation where an allogeneic tissue escapes rejection despite extensive contact with the host. Data from mouse models suggest that maternal T cells recognize fetal antigens through the indirect mechanism, indicating that T cell response results from the uptake and processing of fetal antigen by maternal APCs (118). However, in the context of microchimerism, it is valid to speculate that a T cell response could be also induced by antigen presentation by migratory fetal cells themselves or by the shedding of fetal MHC complexes and their subsequent uptake and retention as intact molecules on the cell surface of maternal APCs (5, 119).

Recently, it has been demonstrated that intact antigens can be transferred between different cell types including T cells, macrophages, B cells and DCs. DCs can capture and retain unprocessed antigen and can transfer it to naive B cells to initiate a specific response (120, 121). This evidence raises the possibility that presentation and recognition of intact alloantigens may also occur on the surface of host APCs without the use of the so called classical mechanisms. In this *semi-direct* allorecognition, allogeneic MHC molecules from the graft (potentially the fetus) are acquired by host APCs, where they are not processed to allopeptides, but integrated in the membrane as conformationally intact MHC complex presenting graft antigens. This phenomenon is also known as membrane alloantigen acquisition or cross-dressing depending on the cell types involved in the exchange and the experimental model (122–124). Although host T cells recognize “intact” graft antigens, this is discussed as a distinct pathway. This results in activation of the same T cell clones as those which respond *via* direct pathway allorecognition (125). However, the mechanisms by which intact MHC-alloantigen complexes are transferred between cells remain poorly understood.

In the field of transplantation, early studies have suggested that cell-to-cell contact is required for host cells to acquire foreign antigens, but recent publications have proposed additional mechanisms involving EVs (126, 127). These are based on the observations that despite finding only very few and in some cases no donor cells in the lymph nodes of mice after skint, heart or islet transplantation, many host cells can be found therein cross-dressed with donor MHC molecules (127). Donor EVs may represent here a source of donor MHC for T (including Treg) cell activation suggesting an additional role for EVs in the generation of an immune response against the allograft (126, 127). The intact foreign MHC molecules pre-loaded with antigen-derived peptides that are integrated into the surface of murine host DCs after uptake of graft-derived EVs, can be later recognized by host T cells, which is in essence, semi-direct allorecognition (**Figure 2** middle panel) (120, 121). An additional mechanism of EV-mediated allorecognition, which does not require intracellular processing, is based on the confocal microscopy observation of a fraction of exosomes secreted by migrating mouse DCs that remain at least 4h as clusters on the surface of conventional host DCs in lymphoid organs (126). In dendritic cells, this ability to retain most exosomes on the surface is characteristic of mature but not immature cells (128). With the appropriate orientation, the donor EVs may provide the MHC complex whereas the host APCs provide the required T-cell costimulatory molecules allowing the presentation without further processing to stimulate T cells (**Figure 2** lower panel) (116).The capacity of mature DCs to retain EVs organized in clusters at the surface may prevent the dilution of the specific MHC complexes and support the formation of functional immune synapses (116). This mechanism of cross-dressing has not been confirmed yet in the context of human pregnancy but some studies including ours have provided evidence of clusters of trophoblast- and placental-derived EVs attached to immune cells (107, 109, 114).

Maternal microchimerism can generate a so-called “split tolerance” in the offspring. This term has two different meanings: it may refer to simultaneous acceptance or rejection of 2 different tissues or organs from one donor or to divers reactions of different components of the immune system on the same antigen (129, 130). In pregnancy, this occurs as either an effector or a tolerogenic response in T cells depending on the origin of the antigen-presenting molecules and the presence of costimulatory factors (43). The different response to maternal microchimeric cells in a mouse model was explained through two potential mechanisms: In the first one, allomolecules from maternal microchimeric cells are released as soluble factors or *via* EVs an taken up by fetal DCs. After intracellular processing, antigens can be presented to fetal T cells in a self-MHC–restricted manner inducing the indirect pathway of allorecognition (43). In the second one, EV membranes containing costimulatory factors and allo-MHC molecules presenting alloantigens are integrated in fetal DC and activate T cells. Depending on the molecules and costimulatory factors present in the antigenic microdomains, either regulatory or effector T cell clones will be specifically induced, driving to split tolerance (43). Similarly, fetal microchimeric IPA+ cells may exert an allorecognition mechanism mediated by fetal-derived EVs carrying HLA-G complexes. However, there is no evidence for cross-dressing mechanism *via* EVs in the context of maternal immunotolerance to fetal cells yet.

## EVs as Enhancers of Tropism of Microchimeric Cells

Metastasizing is the process by which cancer cells are dispersed from the primary tumor and travel *via* blood or lymph system to form a new tumor in other tissues (131). It includes a cascade of separate events that begins with disruption of the original microenvironment, local invasion through extracellular matrix and stromal cell layers, intravasation into the lumina of blood vessels, survival in blood circulation, and subsequent arrest at distant organ sites where they extravasate into the parenchyma. Finally, metastatic cells re-start their proliferative features generating macroscopic, clinically detectable neoplastic growths, which is referred to as metastatic colonization (132–135). Studies in the field of cancer indicate that tumor-derived EVs play a significant role in the cascade of metastasis and can induce the formation of the pre-metastatic niche strengthening the tropism and establishment of new tumors (81, 136, 137).

**Figure 3:**
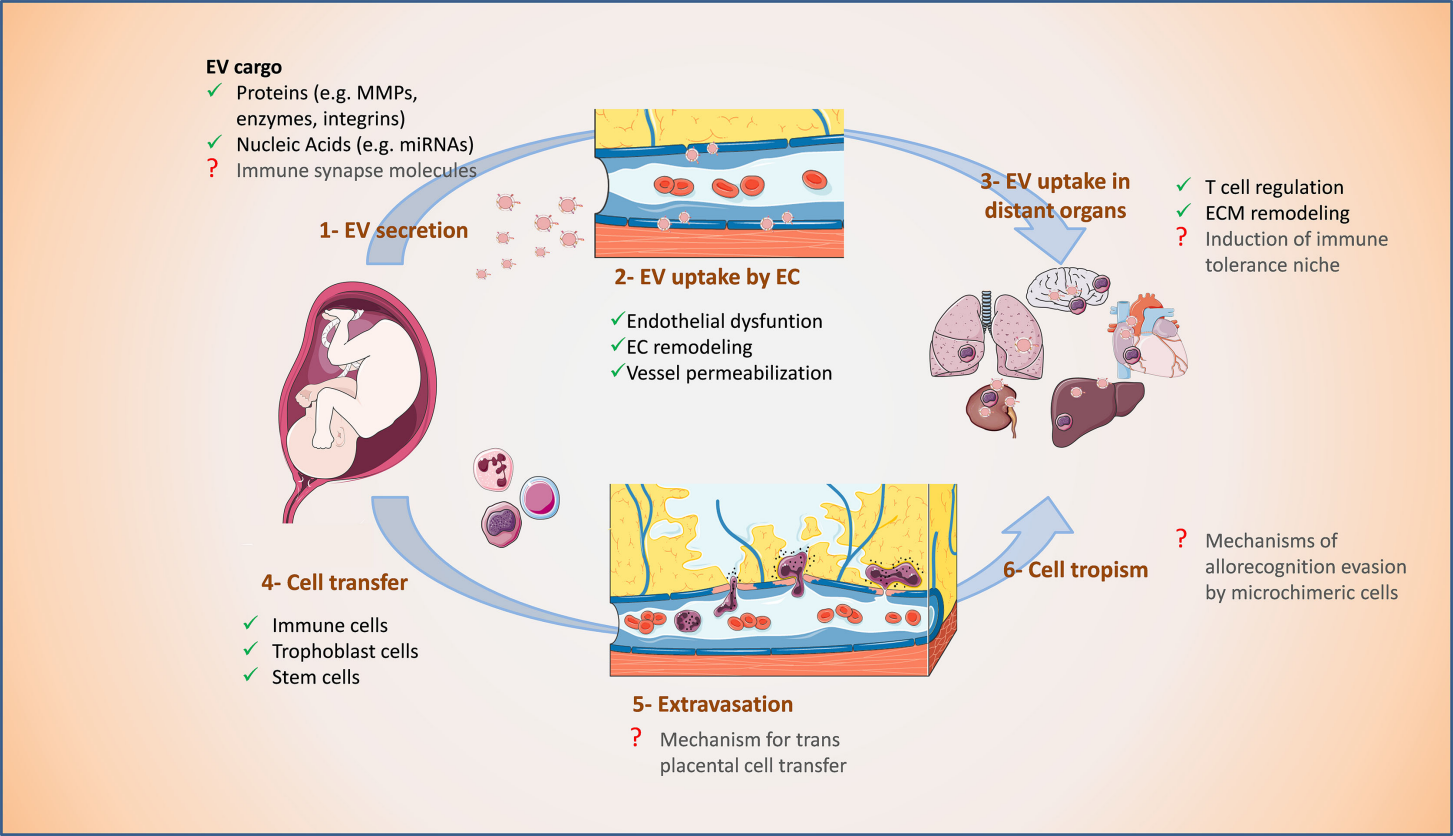
Proposed supportive effect of fetal EVs on microchimerism during pregnancy. 1. Fetal EVs are secreted mainly by the placenta and reach the maternal circulation. 2. Fetal EVs are taken up by endothelial cells (EC), alter their morphology and function, and cause vessel permeabilization. 3. EVs can also reach distant organs, influence their immune cell environment and induce changes in their extracellular matrix (ECM). 4. Fetal cells can trespass the placental barrier and access maternal vessels. 5. Profiting from the EC alterations, fetal cells can extravasate maternal vessels and 6. Access the parenchyma of distant organs as microchimeric cells. Processes indicated with a red question mark are yet to be confirmed. The figure was drawn using pictures from 105 (http://smart.servier.com/).

The pre-metastatic niche is a preformed microenvironment prepared for the colonization and dissemination of cancer cells in specific organs (138, 139). It is characterized by immunosuppression as well as enhanced inflammation, angiogenesis, and vascular permeability (140, 141). EVs released by primary tumors contain proteins and other active molecules which, after internalization by cells in secondary organs, can alter these processes to generate a supportive microenvironment prior to widespread metastasis (81, 136, 137, 142). Analog to tumor-derived EVs, the specific surface and composition of fetal-derived EVs may promote cell trafficking and may induce in distant maternal organs an immunotolerant niche that allows the establishment of microchimerism (**Figure 3**).

EVs from placenta and their tumoral counterparts are similar in their cargo. For instance, several miRNAs known as regulators of angiogenesis, EMT, and invasion in cancer were also reported in placenta- and trophoblast-derived EVs (107, 143–145). Transfer of this miRNA cargo to endothelial cells can cause endothelial dysfunction by inducing oxidative stress and membrane damage. In breast cancer, EVs carrying miR-105 cause destruction of tight junction protein ZO-1 in recipient endothelial cells which increases vascular permeability and susceptibility for metastatic invasion (136). Exposure to EVs from first trimester human placenta affect endothelium-dependent vasodilation of mesenteric arteries in pregnant mice (146). Further, STB-derived EVs transfer miRNAs to endothelial cells affecting target gene expression therein and in pregnancy pathologies such as PE, STB-derived EVs cause extensive cell membrane damage (104). This suggests that placental EVs participate in the regulation of maternal vascular adaptations in pregnancy which is relevant for the trafficking of cells across the endothelial barriers (**Figure 3**).

EV content can also be affected by additional conditions such as stress stimuli. Hypoxia-derived sEVs from human prostate cancer cells are loaded with increased amounts of TGF-β, TNF1-α, and IL-6 and have elevated matrix metalloproteinase (MMP)-2/9 activity. These sEVs induce expression of ECM proteins, and promote CD11b^+^ cells at selected organ sites (147). During the first trimester of pregnancy, a hypoxic environment is maintained in the placenta. In response to low oxygen tension, the release of sEVs from trophoblastic cells (148) and first-trimester primary trophoblast cells (149) increases. Under hypoxic conditions, trophoblastic EVs contain a specific set of miRNAs that could potentially target genes involved in cell migration and inflammatory responses (143, 148). Uptake of these EVs may facilitate or impede vascular remodeling by inducing changes in cell migration and TNF-α secretion of endothelial cells (148).

Cancer- and trophoblast-derived EVs also share their potential to induce immunotolerance and cell migration. EVs derived from metastatic melanomas carry programmed death 1-ligand 1 (PD-L1) on their surface which interacts with programmed cell death protein 1 (PD-1) on CD8^+^ T cells and induces their inactivation. Stimulation of melanoma cells with IFN-γ increases the amount of PD-L1 on the surface of sEVs enhancing CD8^+^ T cell suppression and facilitating tumor growth (150–152). Akin to cancer EVs, those secreted by trophoblastic cells and placenta explants may modulate the immune environment by regulating, either positively or negatively, proliferation and apoptosis of T cells (107, 113, 153). EVs derived from trophoblast cells also modulate cytokine secretion and migration of macrophages, which may contribute to the immunoregulation of the recipient tissues (154, 155). Further, sEVs containing activated matrix MMP-2 2 derived from cancer cells modulate extracellular matrix by degrading collagen and fibronectin to promote cell invasion and metastasis (156). Comparably, EVs derived from trophoblast spheroids can modulate major pathways in endothelial cells including extracellular matrix organization (157).

The membrane of tumor-derived EVs expresses protein “zip-codes”, formed by specific integrin profiles, addressing specific target organs, and thus, determining metastatic organotropism (158). This may also occur during pregnancy and may explain how tropism can be directed to specific organs. Although this area is still largely unknown, a recent study has demonstrated that exogenously administered pregnancy-associated EVs traffic specifically to interstitial lung macrophages and liver Kupffer cells and associate in an integrin-dependent manner (159). Further, 24 h after intravenous injection, human placental sEVs have been localized in the lung, kidney, and liver of mice (146). This preliminary evidence supports a specific EV tropism in pregnancy to maternal organs that are most prone to harbor microchimeric cells, indicating synergies of EV and cell tropism.

## Conclusion and Future Directions

In the last decades, based on the observations of Peter Medawar and other scientists, several mechanisms employed by fetal cells to induce immunotolerance in the mother have been elucidated. The findings summarized in this review suggest that pregnancy-induced microchimerism and exchange of EVs play a pivotal role in modulating T cell responses. Recent studies in transplantology have proposed mechanisms linking the acquisition of graft MHC class II antigens through microchimerism and EVs, with induction of immunotolerance, and in oncology, with preparation of immunotolerant niches in distant organs to accept metastases. In the context of pregnancy, similar mechanisms may occur to induce or support immunotolerance between mother and fetus. These include tolerogenic signals contained in EVs or antigen presentation mediated by microchimeric cells and EVs, as well as the preparation of niches for subsequent intentional or incidental acceptance of microchimeric cells. There is evidence for some of these mechanisms, mostly in the fetus-to-mother direction, but more studies are needed to confirm they occurrence and relevance in the bidirectional communication. Understanding these processes in pregnancy is of relevance as they are potentially altered or involved in pathologies. Further, novel technologies to modify surface and cargo of fetal EVs may selectively influence maternal APC *via* uptake or co-dressing and lead to the development of novel therapeutic strategies.

## Author Contributions

JM-C and DM-P contributed to conceptualization. JM-C, PF-Z, SO-P, and DM-P wrote the first draft. JM-C, PF-Z, and DM-P drafted the manuscript figures. UM participated in correcting, and critically editing the first draft. DM-P, JM-C, and UM edited and approved the final version of the manuscript. All authors approved the submitted version.

## Funding

DM-P and UM have been supported by the German Research Foundation (DFG, grant Mo2017/3-2 and Mo2017/3-3 to DM-P and Ma1550/12-1 to UM) and the Interdisciplinary Center for Clinical Research (IZKF, DMMP FF05) at the Jena University Hospital. JM-C has received a postgraduate grant from CONACyT (CVU: 446429). PF-Z receives a Ph.D. scholarship (Personal Reference No. 91771964) from the German Academic Exchange Service (DAAD).

## Conflict of Interest

The authors declare that the research was conducted in the absence of any commercial or financial relationships that could be construed as a potential conflict of interest.

## Publisher’s Note

All claims expressed in this article are solely those of the authors and do not necessarily represent those of their affiliated organizations, or those of the publisher, the editors and the reviewers. Any product that may be evaluated in this article, or claim that may be made by its manufacturer, is not guaranteed or endorsed by the publisher.

## References

[1] Medawar PB. Peter Medawar – Nobel Lecture. NobelPrize.org. Nobel Prize Outreach AB 2022 (1960). Available at: https://www.nobelprize.org/prizes/medicine/1960/medawar/lecture/ (Accessed 2022).

[2] Medawar PB. Immunological Tolerance. Nature (1961) 189:14–7. doi: 10.1038/189014a0 13768821

[3] Male V. Medawar and the Immunological Paradox of Pregnancy: In Context. Oxford Open Immunol (2020) 2(1):iqaa006. doi: 10.1093/oxfimm/iqaa006 PMC9914476 36845570

[4] Rendell V Bath NM Brennan TV. Medawar’s Paradox and Immune Mechanisms of Fetomaternal Tolerance. OBM Transplant (2020) 4(1):26. doi: 10.21926/obm.transplant.2001104 32582882 PMC7314236

[5] Bracamonte-Baran W Burlingham W. Non-Inherited Maternal Antigens, Pregnancy, and Allotolerance. BioMed J (2015) 38:39–51. doi: 10.4103/2319-4170.143498 25355389

[6] Chen JC. Immunological Consequences of *In Utero* Exposure to Foreign Antigens. Front Immunol (2021) 12:638435. doi: 10.3389/fimmu.2021.638435 33936052 PMC8082100

[7] Owen RD. Immunogenetic Consequences of Vascular Anastomoses Between Bovine Twins. Science (1945) 102:400–1. doi: 10.1126/science.102.2651.400 17755278

[8] Owen RD Wood HR Foord AG Sturgeon P Baldwin LG. EVIDENCE FOR ACTIVELY ACQUIRED TOLERANCE TO Rh ANTIGENS. Proc Natl Acad Sci U.S.A. (1954) 40:420–4. doi: 10.1073/pnas.40.6.420. PMC534062 16589498

[9] Burnet FM Fenner F. Production of Antibodies. Nature (1950) 166:204–5. doi: 10.1038/166204a0

[10] Martin A. Ray Owen and the History of Naturally Acquired Chimerism. Chimerism (2015) 6:2–7. doi: 10.1080/19381956.2016.1168561 27093621 PMC5063071

[11] Billingham RE Brent L Medawar PB. Actively Acquired Tolerance of Foreign Cells. Nature (1953) 172:603–6. doi: 10.1038/172603a0 13099277

[12] Dunsford I Bowley CC Hutchison AM Thompson JS Sanger R Race RR. A Human Blood-Group Chimera. Br Med J (1953) 2:81. doi: 10.1136/bmj.2.4827.81 13051584 PMC2028470

[13] Martin A. ‘Incongruous Juxtapositions’: The Chimaera and Mrs McK. Endeavour (2007) 31:99–103. doi: 10.1016/j.endeavour.2007.07.003 17727951

[14] Anderson D Billingham RE Lampkin GH Medawar PB. The Use of Skin Grafting to Distinguish Between Monozygotic and Dizygotic Twins in Cattle. Heredity (1951) 5:379–97. doi: 10.1038/hdy.1951.38

[15] Shrivastava S Naik R Suryawanshi H Gupta N. Microchimerism: A New Concept. J Oral Maxillofac Pathol (2019) 23:311. doi: 10.4103/jomfp.JOMFP_85_17 PMC6714269 31516258

[16] Gammill HS Harrington WE. Microchimerism: Defining and Redefining the Prepregnancy Context - A Review. Placenta (2017) 60:130–3. doi: 10.1016/j.placenta.2017.08.071 PMC5718967 28911790

[17] Hahn S Hasler P Vokalova L Van Breda SV Than NG Hoesli IM. Feto-Maternal Microchimerism: The Pre-Eclampsia Conundrum. Front Immunol (2019) 10:659. doi: 10.3389/fimmu.2019.00659 31001268 PMC6455070

[18] Murrieta-Coxca JM Aengenheister L Schmidt A Markert UR Buerki-Thurnherr T Morales-Prieto DM. Addressing Microchimerism in Pregnancy by Ex Vivo Human Placenta Perfusion. Placenta (2021) 117:78–86. doi: 10.1016/j.placenta.2021.10.004 34773744

[19] Thomas MR Tutschek B Frost A Rodeck CH Yazdani N Craft I. The Time of Appearance and Disappearance of Fetal DNA From the Maternal Circulation. Prenat Diagn (1995) 15:641–6. doi: 10.1002/pd.1970150709 8532624

[20] Pertl B Bianchi DW. First Trimester Prenatal Diagnosis: Fetal Cells in the Maternal Circulation. Semin Perinatol (1999) 23:393–402. doi: 10.1016/S0146-0005(99)80005-6 10551792

[21] Lo YM Tein MS Lau TK Haines CJ Leung TN Poon PM. Quantitative Analysis of Fetal DNA in Maternal Plasma and Serum: Implications for Noninvasive Prenatal Diagnosis. Am J Hum Genet (1998) 62:768–75. doi: 10.1086/301800 PMC1377040 9529358

[22] Lo YM Zhang J Leung TN Lau TK Chang AM Hjelm NM. Rapid Clearance of Fetal DNA From Maternal Plasma. Am J Hum Genet (1999) 64:218–24. doi: 10.1086/302205 PMC1377720 9915961

[23] Chargaff E West R. The Biological Significance of the Thromboplastic Protein of Blood. J Biol Chem (1946) 166:189–97. doi: 10.1016/S0021-9258(17)34997-9 20273687

[24] Bianchi DW Zickwolf GK Weil GJ Sylvester S Demaria MA. Male Fetal Progenitor Cells Persist in Maternal Blood for as Long as 27 Years Postpartum. Proc Natl Acad Sci U.S.A. (1996) 93:705–8. doi: 10.1073/pnas.93.2.705. PMC40117 8570620

[25] Maloney S Smith A Furst DE Myerson D Rupert K Evans PC. Microchimerism of Maternal Origin Persists Into Adult Life. J Clin Invest (1999) 104:41–7. doi: 10.1172/JCI6611 PMC408407 10393697

[26] Lambert NC Erickson TD Yan Z Pang JM Guthrie KA Furst DE. Quantification of Maternal Microchimerism by HLA-Specific Real-Time Polymerase Chain Reaction: Studies of Healthy Women and Women With Scleroderma. Arthritis Rheum (2004) 50:906–14. doi: 10.1002/art.20200 15022334

[27] O’donoghue K Chan J de la Fuente J Kennea N Sandison A Anderson JR. Microchimerism in Female Bone Marrow and Bone Decades After Fetal Mesenchymal Stem-Cell Trafficking in Pregnancy. Lancet (2004) 364:179–82. doi: 10.1016/S0140-6736(04)16631-2 15246731

[28] Bayes-Genis A Bellosillo B de la Calle O Salido M Roura S Ristol FS. Identification of Male Cardiomyocytes of Extracardiac Origin in the Hearts of Women With Male Progeny: Male Fetal Cell Microchimerism of the Heart. J Heart Lung Transplant (2005) 24:2179–83. doi: 10.1016/j.healun.2005.06.003 16364868

[29] Kinder JM Stelzer IA Arck PC Way SS. Immunological Implications of Pregnancy-Induced Microchimerism. Nat Rev Immunol (2017) 17:483–94. doi: 10.1038/nri.2017.38 PMC5532073 28480895

[30] Simpson JL. Preimplantation Genetics and Recovery of Fetal Cells From Maternal Blood. Curr Opin Obstet Gynecol (1992) 4:295–301. doi: 10.1097/00001703-199204000-00016 1571491

[31] Lo YM Lau TK Chan LY Leung TN Chang AM. Quantitative Analysis of the Bidirectional Fetomaternal Transfer of Nucleated Cells and Plasma DNA. Clin Chem (2000) 46:1301–9. doi: 10.1093/clinchem/46.9.1301 10973858

[32] Gammill HS Nelson JL. Naturally Acquired Microchimerism. Int J Dev Biol (2010) 54:531–43. doi: 10.1387/ijdb.082767hg PMC2887685 19924635

[33] Lapaire O Holzgreve W Oosterwijk JC Brinkhaus R Bianchi DW. Georg Schmorl on Trophoblasts in the Maternal Circulation. Placenta (2007) 28:1–5. doi: 10.1016/j.placenta.2006.02.004 16620961

[34] Khosrotehrani K Johnson KL Guegan S Stroh H Bianchi DW. Natural History of Fetal Cell Microchimerism During and Following Murine Pregnancy. J Reprod Immunol (2005) 66:1–12. doi: 10.1016/j.jri.2005.02.001 15949558

[35] Dawe GS Tan XW Xiao ZC. Cell Migration From Baby to Mother. Cell Adh Migr (2007) 1:19–27. doi: 10.4161/cam.4082 19262088 PMC2633676

[36] Stelzer IA Thiele K Solano ME. Maternal Microchimerism: Lessons Learned From Murine Models. J Reprod Immunol (2015) 108:12–25. doi: 10.1016/j.jri.2014.12.007 25638482

[37] Rijnink EC Penning ME Wolterbeek R Wilhelmus S Zandbergen M Van Duinen SG. Tissue Microchimerism is Increased During Pregnancy: A Human Autopsy Study. Mol Hum Reprod (2015) 21:857–64. doi: 10.1093/molehr/gav047 26307194

[38] Jonsson AM Uzunel M Gotherstrom C Papadogiannakis N Westgren M. Maternal Microchimerism in Human Fetal Tissues. Am J Obstet Gynecol (2008) 198:325.e321–326. doi: 10.1016/j.ajog.2007.09.047 18191801

[39] Dutta P Molitor-Dart M Bobadilla JL Roenneburg DA Yan Z Torrealba JR. Microchimerism is Strongly Correlated With Tolerance to Noninherited Maternal Antigens in Mice. Blood (2009) 114:3578–87. doi: 10.1182/blood-2009-03-213561 PMC2766676 19700665

[40] Dutta P Burlingham WJ. Stem Cell Microchimerism and Tolerance to non-Inherited Maternal Antigens. Chimerism (2010) 1:2–10. doi: 10.4161/chim.1.1.12667 21132055 PMC2995256

[41] Dutta P Burlingham WJ. Correlation Between Post Transplant Maternal Microchimerism and Tolerance Across MHC Barriers in Mice. Chimerism (2011) 2:78–83. doi: 10.4161/chim.18083 22163065 PMC3234359

[42] Anderson CC Matzinger P. Immunity or Tolerance: Opposite Outcomes of Microchimerism From Skin Grafts. Nat Med (2001) 7:80–7. doi: 10.1038/83393 11135620

[43] Bracamonte-Baran W Florentin J Zhou Y Jankowska-Gan E Haynes WJ Zhong W. Modification of Host Dendritic Cells by Microchimerism-Derived Extracellular Vesicles Generates Split Tolerance. Proc Natl Acad Sci U.S.A. (2017) 114:1099–104. doi: 10.1073/pnas.1618364114 PMC5293109 28096390

[44] Federici BA Roberts DW. Experimental Laboratory Infection of Mosquito Larvae With Fungi of the Genus Coelomomyces. 1. Experiments With Coelomomyces Psorophorae Var. In Aedes Taeniorhynchus and Coelomomyces Psorophorae Var. In Culiseta Inornata. J Invertebr Pathol (1975) 26:21–7. doi: 10.1016/0022-2011(75)90164-0 239068

[45] Mor G Cardenas I Abrahams V Guller S. Inflammation and Pregnancy: The Role of the Immune System at the Implantation Site. Ann N Y Acad Sci (2011) 1221:80–7. doi: 10.1111/j.1749-6632.2010.05938.x PMC3078586 21401634

[46] Arck PC Hecher K. Fetomaternal Immune Cross-Talk and its Consequences for Maternal and Offspring’s Health. Nat Med (2013) 19:548–56. doi: 10.1038/nm.3160 23652115

[47] Blois SM Joachim R Kandil J Margni R Tometten M Klapp BF. Depletion of CD8+ Cells Abolishes the Pregnancy Protective Effect of Progesterone Substitution With Dydrogesterone in Mice by Altering the Th1/Th2 Cytokine Profile. J Immunol (2004) 172:5893–9. doi: 10.4049/jimmunol.172.10.5893 15128769

[48] Tilburgs T Roelen DL van der Mast BJ Van Schip JJ Kleijburg C De Groot-Swings GM. Differential Distribution of CD4(+)CD25(bright) and CD8(+)CD28(-) T-Cells in Decidua and Maternal Blood During Human Pregnancy. Placenta (2006) 27 Suppl A:S47–53. doi: 10.1016/j.placenta.2005.11.008 16442616

[49] Norton MT Fortner KA Oppenheimer KH Bonney EA. Evidence That CD8 T-Cell Homeostasis and Function Remain Intact During Murine Pregnancy. Immunology (2010) 131:426–37. doi: 10.1111/j.1365-2567.2010.03316.x PMC2996563 20553337

[50] Rieger L Segerer S Bernar T Kapp M Majic M Morr AK. Specific Subsets of Immune Cells in Human Decidua Differ Between Normal Pregnancy and Preeclampsia–a Prospective Observational Study. Reprod Biol Endocrinol (2009) 7:132. doi: 10.1186/1477-7827-7-132 19930648 PMC2789084

[51] Apps R Murphy SP Fernando R Gardner L Ahad T Moffett A. Human Leucocyte Antigen (HLA) Expression of Primary Trophoblast Cells and Placental Cell Lines, Determined Using Single Antigen Beads to Characterize Allotype Specificities of Anti-HLA Antibodies. Immunology (2009) 127:26–39. doi: 10.1111/j.1365-2567.2008.03019.x 19368562 PMC2678179

[52] Le Gal FA Riteau B Sedlik C Khalil-Daher I Menier C Dausset J. HLA-G-Mediated Inhibition of Antigen-Specific Cytotoxic T Lymphocytes. Int Immunol (1999) 11:1351–6. doi: 10.1093/intimm/11.8.1351 10421792

[53] Kshirsagar SK Alam SM Jasti S Hodes H Nauser T Gilliam M. Immunomodulatory Molecules are Released From the First Trimester and Term Placenta *via* Exosomes. Placenta (2012) 33:982–90. doi: 10.1016/j.placenta.2012.10.005 PMC3534832 23107341

[54] Poehlmann TG Schaumann A Busch S Fitzgerald JS Aguerre-Girr M Le Bouteiller P. Inhibition of Term Decidual NK Cell Cytotoxicity by Soluble HLA-G1. Am J Reprod Immunol (2006) 56:275–85. doi: 10.1111/j.1600-0897.2006.00420.x 17076671

[55] Nair S Salomon C. Extracellular Vesicles and Their Immunomodulatory Functions in Pregnancy. Semin Immunopathol (2018) 40:425–37. doi: 10.1007/s00281-018-0680-2 29616307

[56] Mold JE Michaelsson J Burt TD Muench MO Beckerman KP Busch MP. Maternal Alloantigens Promote the Development of Tolerogenic Fetal Regulatory T Cells *In Utero* . Science (2008) 322:1562–5. doi: 10.1126/science.1164511 PMC2648820 19056990

[57] Samstein RM Josefowicz SZ Arvey A Treuting PM Rudensky AY. Extrathymic Generation of Regulatory T Cells in Placental Mammals Mitigates Maternal-Fetal Conflict. Cell (2012) 150:29–38. doi: 10.1016/j.cell.2012.05.031 22770213 PMC3422629

[58] Hazes JM Dijkmans BA Vandenbroucke JP De Vries RR Cats A. Pregnancy and the Risk of Developing Rheumatoid Arthritis. Arthritis Rheum (1990) 33:1770–5. doi: 10.1002/art.1780331203 2260999

[59] Masera S Cavalla P Prosperini L Mattioda A Mancinelli CR Superti G. Parity is Associated With a Longer Time to Reach Irreversible Disability Milestones in Women With Multiple Sclerosis. Mult Scler (2015) 21:1291–7. doi: 10.1177/1352458514561907 25533293

[60] Santos MA O’donoghue K Wyatt-Ashmead J Fisk NM. Fetal Cells in the Maternal Appendix: A Marker of Inflammation or Fetal Tissue Repair? Hum Reprod (2008) 23:2319–25. doi: 10.1093/humrep/den261 18617594

[61] Sunami R Komuro M Yuminamochi T Hoshi K Hirata S. Fetal Cell Microchimerism Develops Through the Migration of Fetus-Derived Cells to the Maternal Organs Early After Implantation. J Reprod Immunol (2010) 84:117–23. doi: 10.1016/j.jri.2009.11.006 20116109

[62] Zeng XX Tan KH Yeo A Sasajala P Tan X Xiao ZC. Pregnancy-Associated Progenitor Cells Differentiate and Mature Into Neurons in the Maternal Brain. Stem Cells Dev (2010) 19:1819–30. doi: 10.1089/scd.2010.0046 20707697

[63] Kara RJ Bolli P Karakikes I Matsunaga I Tripodi J Tanweer O. Fetal Cells Traffic to Injured Maternal Myocardium and Undergo Cardiac Differentiation. Circ Res (2012) 110:82–93. doi: 10.1161/CIRCRESAHA.111.249037 22082491 PMC3365532

[64] Mahmood U O’donoghue K. Microchimeric Fetal Cells Play a Role in Maternal Wound Healing After Pregnancy. Chimerism (2014) 5:40–52. doi: 10.4161/chim.28746 24717775 PMC4199806

[65] Roy E Seppanen E Ellis R Lee ES Khosrotehrani K Fisk NM. Biphasic Recruitment of Microchimeric Fetal Mesenchymal Cells in Fibrosis Following Acute Kidney Injury. Kidney Int (2014) 85:600–10. doi: 10.1038/ki.2013.459 24304884

[66] Goldman AS Garza C Nichols BL Goldblum RM. Immunologic Factors in Human Milk During the First Year of Lactation. J Pediatr (1982) 100:563–7. doi: 10.1016/S0022-3476(82)80753-1 6977634

[67] Boddy AM Fortunato A Wilson Sayres M Aktipis A. Fetal Microchimerism and Maternal Health: A Review and Evolutionary Analysis of Cooperation and Conflict Beyond the Womb. Bioessays (2015) 37:1106–18. doi: 10.1002/bies.201500059 PMC4712643 26316378

[68] Touzot F Dal-Cortivo L Verkarre V Lim A Crucis-Armengaud A Moshous D. Massive Expansion of Maternal T Cells in Response to EBV Infection in a Patient With SCID-Xl. Blood (2012) 120:1957–9. doi: 10.1182/blood-2012-04-426833 22936741

[69] Chargaff E. CELL STRUCTURE AND THE PROBLEM OF BLOOD COAGULATION. J Biol Chem (1945) 160:351–9. doi: 10.1016/S0021-9258(18)43131-6

[70] Wolf P. The Nature and Significance of Platelet Products in Human Plasma. Br J Haematol (1967) 13:269–88. doi: 10.1111/j.1365-2141.1967.tb08741.x 6025241

[71] Crawford N. The Presence of Contractile Proteins in Platelet Microparticles Isolated From Human and Animal Platelet-Free Plasma. Br J Haematol (1971) 21:53–69. doi: 10.1111/j.1365-2141.1971.tb03416.x 4254312

[72] Nunez EA Wallis J Gershon MD. Secretory Processes in Follicular Cells of the Bat Thyroid. 3. The Occurrence of Extracellular Vesicles and Colloid Droplets During Arousal From Hibernation. Am J Anat (1974) 141:179–201. doi: 10.1002/aja.1001410203 4415703

[73] Harding C Heuser J Stahl P. Receptor-Mediated Endocytosis of Transferrin and Recycling of the Transferrin Receptor in Rat Reticulocytes. J Cell Biol (1983) 97:329–39. doi: 10.1083/jcb.97.2.329 PMC2112509 6309857

[74] Trams EG Lauter CJ Salem N Heine U. Exfoliation of Membrane Ecto-Enzymes in the Form of Micro-Vesicles. Biochim Biophys Acta (1981) 645:63–70. doi: 10.1016/0005-2736(81)90512-5 6266476

[75] Pan BT Johnstone RM. Fate of the Transferrin Receptor During Maturation of Sheep Reticulocytes *In Vitro*: Selective Externalization of the Receptor. Cell (1983) 33:967–78. doi: 10.1016/0092-8674(83)90040-5 6307529

[76] Smith NC Brush MG Luckett S. Preparation of Human Placental Villous Surface Membrane. Nature (1974) 252:302–3. doi: 10.1038/252302b0 4431448

[77] Mcintyre JA Faulk WP. Trophoblast Modulation of Maternal Allogeneic Recognition. Proc Natl Acad Sci U.S.A. (1979) 76:4029–32. doi: 10.1073/pnas.76.8.4029. PMC383970 158764

[78] Goodfellow CF Booth AG. The Actions of Composite Trophoblast Antigens in Microvillus Preparations Upon Cultured Maternal Lymphocytes From Early First Pregnancies. Eur J Obstetrics Gynecology Reprod Biol (1982) 13:15–21. doi: 10.1016/0028-2243(82)90033-8 7060814

[79] Knight M Redman CW Linton EA Sargent IL. Shedding of Syncytiotrophoblast Microvilli Into the Maternal Circulation in Pre-Eclamptic Pregnancies. Br J Obstet Gynaecol (1998) 105:632–40. doi: 10.1111/j.1471-0528.1998.tb10178.x 9647154

[80] Thery C Witwer KW Aikawa E Alcaraz MJ Anderson JD Andriantsitohaina R. Minimal Information for Studies of Extracellular Vesicles 2018 (MISEV2018): A Position Statement of the International Society for Extracellular Vesicles and Update of the MISEV2014 Guidelines. J Extracell Vesicles (2018) 7:1535750. doi: 10.1080/20013078.2018.1535750 30637094 PMC6322352

[81] Kogure A Yoshioka Y Ochiya T. Extracellular Vesicles in Cancer Metastasis: Potential as Therapeutic Targets and Materials. Int J Mol Sci (2020) 21(12): 4463. doi: 10.3390/ijms21124463 PMC7352700 32585976

[82] Lobb RJ Lima LG Moller A. Exosomes: Key Mediators of Metastasis and Pre-Metastatic Niche Formation. Semin Cell Dev Biol (2017) 67:3–10. doi: 10.1016/j.semcdb.2017.01.004 28077297

[83] Malkin EZ Bratman SV. Bioactive DNA From Extracellular Vesicles and Particles. Cell Death Dis (2020) 11:584. doi: 10.1038/s41419-020-02803-4 32719324 PMC7385258

[84] Elzanowska J Semira C Costa-Silva B. DNA in Extracellular Vesicles: Biological and Clinical Aspects. Mol Oncol (2021) 15:1701–14. doi: 10.1002/1878-0261.12777 PMC8169445 32767659

[85] Gould SJ Raposo G. As We Wait: Coping With an Imperfect Nomenclature for Extracellular Vesicles. J Extracell Vesicles (2013) 2:10.3402/jev.v2i0.20389. doi: 10.3402/jev.v2i0.20389 PMC3760635 24009890

[86] Colombo M Raposo G Thery C. Biogenesis, Secretion, and Intercellular Interactions of Exosomes and Other Extracellular Vesicles. Annu Rev Cell Dev Biol (2014) 30:255–89. doi: 10.1146/annurev-cellbio-101512-122326 25288114

[87] Yanez-Mo M Siljander PR Andreu Z Zavec AB Borras FE Buzas EI. Biological Properties of Extracellular Vesicles and Their Physiological Functions. J Extracell Vesicles (2015) 4:27066. doi: 10.3402/jev.v4.27066 25979354 PMC4433489

[88] Göhner C Weber M Tannetta DS Groten T Plösch T Faas MM. A New Enzyme-Linked Sorbent Assay (ELSA) to Quantify Syncytiotrophoblast Extracellular Vesicles in Biological Fluids. Am J Reprod Immunol (2015) 73:582–8. doi: 10.1111/aji.12367 25753333

[89] Vargas A Zhou S Éthier-Chiasson M Flipo D Lafond J Gilbert C. Syncytin Proteins Incorporated in Placenta Exosomes are Important for Cell Uptake and Show Variation in Abundance in Serum Exosomes From Patients With Preeclampsia. FASEB J (2014) 28:3703–19. doi: 10.1096/fj.13-239053 24812088

[90] Pap E Pallinger E Falus A Kiss AA Kittel A Kovacs P. T Lymphocytes are Targets for Platelet- and Trophoblast-Derived Microvesicles During Pregnancy. Placenta (2008) 29:826–32. doi: 10.1016/j.placenta.2008.06.006 18684502

[91] Raiborg C Stenmark H. The ESCRT Machinery in Endosomal Sorting of Ubiquitylated Membrane Proteins. Nature (2009) 458:445–52. doi: 10.1038/nature07961 19325624

[92] Van Niel G Charrin S Simoes S Romao M Rochin L Saftig P. The Tetraspanin CD63 Regulates ESCRT-Independent and -Dependent Endosomal Sorting During Melanogenesis. Dev Cell (2011) 21:708–21. doi: 10.1016/j.devcel.2011.08.019 PMC3199340 21962903

[93] Liu H Kang M Wang J Blenkiron C Lee A Wise M. Estimation of the Burden of Human Placental Micro- and Nano-Vesicles Extruded Into the Maternal Blood From 8 to 12 Weeks of Gestation. Placenta (2018) 72-73:41–7. doi: 10.1016/j.placenta.2018.10.009 30501880

[94] Chamley LW Chen Q Ding J Stone PR Abumaree M. Trophoblast Deportation: Just a Waste Disposal System or Antigen Sharing? J Reprod Immunol (2011) 88:99–105. doi: 10.1016/j.jri.2011.01.002 21334749

[95] Wei J Blenkiron C Tsai P James JL Chen Q Stone PR. Placental Trophoblast Debris Mediated Feto-Maternal Signalling *via* Small RNA Delivery: Implications for Preeclampsia. Sci Rep (2017) 7:14681. doi: 10.1038/s41598-017-14180-8 29089639 PMC5665858

[96] Redman CW Sargent IL. Microparticles and Immunomodulation in Pregnancy and Pre-Eclampsia. J Reprod Immunol (2007) 76:61–7. doi: 10.1016/j.jri.2007.03.008 17482271

[97] Hedlund M Stenqvist AC Nagaeva O Kjellberg L Wulff M Baranov V. Human Placenta Expresses and Secretes NKG2D Ligands *via* Exosomes That Down-Modulate the Cognate Receptor Expression: Evidence for Immunosuppressive Function. J Immunol (2009) 183:340–51. doi: 10.4049/jimmunol.0803477 19542445

[98] Tong M Abrahams VM Chamley LW. Immunological Effects of Placental Extracellular Vesicles. Immunol Cell Biol (2018). doi: 10.1111/imcb.12049 29604098

[99] James-Allan LB Rosario FJ Barner K Lai A Guanzon D Mcintyre HD. Regulation of Glucose Homeostasis by Small Extracellular Vesicles in Normal Pregnancy and in Gestational Diabetes. FASEB J (2020) 34:5724–39. doi: 10.1096/fj.201902522RR 32154621

[100] Holder B Jones T Sancho Shimizu V Rice TF Donaldson B Bouqueau M. Macrophage Exosomes Induce Placental Inflammatory Cytokines: A Novel Mode of Maternal-Placental Messaging. Traffic (2016) 17:168–78. doi: 10.1111/tra.12352 PMC4738478 26602702

[101] Rice TF Donaldson B Bouqueau M Kampmann B Holder B. Macrophage- But Not Monocyte-Derived Extracellular Vesicles Induce Placental Pro-Inflammatory Responses. Placenta (2018) 69:92–5. doi: 10.1016/j.placenta.2018.07.011 PMC6398160 30213492

[102] Frangsmyr L Baranov V Nagaeva O Stendahl U Kjellberg L Mincheva-Nilsson L. Cytoplasmic Microvesicular Form of Fas Ligand in Human Early Placenta: Switching the Tissue Immune Privilege Hypothesis From Cellular to Vesicular Level. Mol Hum Reprod (2005) 11:35–41. doi: 10.1093/molehr/gah129 15579659

[103] Holland OJ Linscheid C Hodes HC Nauser TL Gilliam M Stone P. Minor Histocompatibility Antigens are Expressed in Syncytiotrophoblast and Trophoblast Debris: Implications for Maternal Alloreactivity to the Fetus. Am J Pathol (2012) 180:256–66. doi: 10.1016/j.ajpath.2011.09.021 PMC3338347 22079431

[104] Cronqvist T Tannetta D Mörgelin M Belting M Sargent I Familari M. Syncytiotrophoblast Derived Extracellular Vesicles Transfer Functional Placental miRNAs to Primary Human Endothelial Cells. Sci Rep (2017) 7:4558. doi: 10.1038/s41598-017-04468-0 28676635 PMC5496854

[105] Servier Medical Art. (2021). Available at: https://smart.servier.com (Accessed November 2021).

[106] Delorme-Axford E Donker RB Mouillet JF Chu T Bayer A Ouyang Y. Human Placental Trophoblasts Confer Viral Resistance to Recipient Cells. Proc Natl Acad Sci U.S.A. (2013) 110:12048–53. https://smart.servier.com/" SMART - Servier Medical ART 10.1073/pnas.1304718110 PMC3718097 23818581

[107] Chaiwangyen W Murrieta-Coxca JM Favaro RR Photini SM Gutierrez-Samudio RN Schleussner E. MiR-519d-3p in Trophoblastic Cells: Effects, Targets and Transfer to Allogeneic Immune Cells *via* Extracellular Vesicles. Int J Mol Sci (2020) 21(10): 3458. doi: 10.3390/ijms21103458 PMC7278925 32422900

[108] Ma Y Schroder DC Nenkov M Rizwan MN Abubrig M Sonnemann J. Epithelial Membrane Protein 2 Suppresses Non-Small Cell Lung Cancer Cell Growth by Inhibition of MAPK Pathway. Int J Mol Sci (2021) 22(6): 2944. doi: 10.3390/ijms22062944 33799364 PMC7999101

[109] Zabel RR Bar C Ji J Schultz R Hammer M Groten T. Enrichment and Characterization of Extracellular Vesicles From Ex Vivo One-Sided Human Placenta Perfusion. Am J Reprod Immunol (2021) 86:e13377. doi: 10.1111/aji.13377 33175429

[110] Czernek L Chworos A Duechler M. The Uptake of Extracellular Vesicles is Affected by the Differentiation Status of Myeloid Cells. Scand J Immunol (2015) 82:506–14. doi: 10.1111/sji.12371 26332303

[111] Abrahams VM Straszewski-Chavez SL Guller S Mor G. First Trimester Trophoblast Cells Secrete Fas Ligand Which Induces Immune Cell Apoptosis. Mol Hum Reprod (2004) 10:55–63. doi: 10.1093/molehr/gah006 14665707

[112] Sabapatha A Gercel-Taylor C Taylor DD. Specific Isolation of Placenta-Derived Exosomes From the Circulation of Pregnant Women and Their Immunoregulatory Consequences. Am J Reprod Immunol (2006) 56:345–55. doi: 10.1111/j.1600-0897.2006.00435.x 17076679

[113] Ospina-Prieto S Chaiwangyen W Herrmann J Groten T Schleussner E Markert UR. MicroRNA-141 is Upregulated in Preeclamptic Placentae and Regulates Trophoblast Invasion and Intercellular Communication. Transl Res (2016) 172:61–72. doi: 10.1016/j.trsl.2016.02.012 27012474

[114] Awoyemi T Motta-Mejia C Zhang W Kouser L White K Kandzija N. Syncytiotrophoblast Extracellular Vesicles From Late-Onset Preeclampsia Placentae Suppress Pro-Inflammatory Immune Response in THP-1 Macrophages. Front Immunol (2021) 12:676056. doi: 10.3389/fimmu.2021.676056 34163477 PMC8215361

[115] Favaro RR Murrieta-Coxca JM Gutierrez-Samudio RN Morales-Prieto DM Markert UR. Immunomodulatory Properties of Extracellular Vesicles in the Dialogue Between Placental and Immune Cells. Am J Reprod Immunol (2021) 85:e13383. doi: 10.1111/aji.13383 33251688

[116] Zeng F Morelli AE. Extracellular Vesicle-Mediated MHC Cross-Dressing in Immune Homeostasis, Transplantation, Infectious Diseases, and Cancer. Semin Immunopathol (2018) 40:477–90. doi: 10.1007/s00281-018-0679-8 PMC6162176 29594331

[117] Gallon L Watschinger B Murphy B Akalin E Sayegh MH Carpenter CB. The Indirect Pathway of Allorecognition. The Occurrence of Self-Restricted T Cell Recognition of Allo-MHC Peptides Early in Acute Renal Allograft Rejection and its Inhibition by Conventional Immunosuppression. Transplantation (1995) 59:612–6. doi: 10.1097/00007890-199502270-00029 7878766

[118] Erlebacher A Vencato D Price KA Zhang D Glimcher LH. Constraints in Antigen Presentation Severely Restrict T Cell Recognition of the Allogeneic Fetus. J Clin Invest (2007) 117:1399–411. doi: 10.1172/JCI28214 PMC1849983 17446933

[119] Adams KM Yan Z Stevens AM Nelson JL. The Changing Maternal “Self” Hypothesis: A Mechanism for Maternal Tolerance of the Fetus. Placenta (2007) 28:378–82. doi: 10.1016/j.placenta.2006.07.003 16934327

[120] Knight SC Iqball S Roberts MS Macatonia S Bedford PA. Transfer of Antigen Between Dendritic Cells in the Stimulation of Primary T Cell Proliferation. Eur J Immunol (1998) 28:1636–44. doi: 10.1002/(SICI)1521-4141(199805)28:05<1636::AID-IMMU1636>3.0.CO;2-9 9603470

[121] Wykes M Pombo A Jenkins C Macpherson GG. Dendritic Cells Interact Directly With Naive B Lymphocytes to Transfer Antigen and Initiate Class Switching in a Primary T-Dependent Response. J Immunol (1998) 161:1313–9. https://www.jimmunol.org/content/161/3/1313 9686593

[122] Harshyne LA Watkins SC Gambotto A Barratt-Boyes SM. Dendritic Cells Acquire Antigens From Live Cells for Cross-Presentation to CTL. J Immunol (2001) 166:3717–23. doi: 10.4049/jimmunol.166.6.3717 11238612

[123] Herrera OB Golshayan D Tibbott R Salcido Ochoa F James MJ Marelli-Berg FM. A Novel Pathway of Alloantigen Presentation by Dendritic Cells. J Immunol (2004) 173:4828–37. doi: 10.4049/jimmunol.173.8.4828 15470023

[124] Zhang QJ Li XL Wang D Huang XC Mathis JM Duan WM. Trogocytosis of MHC-I/peptide Complexes Derived From Tumors and Infected Cells Enhances Dendritic Cell Cross-Priming and Promotes Adaptive T Cell Responses. PLoS One (2008) 3:e3097. doi: 10.1371/journal.pone.0003097 18769733 PMC2518214

[125] Zeng Z Li Y Pan Y Lan X Song F Sun J. Cancer-Derived Exosomal miR-25-3p Promotes Pre-Metastatic Niche Formation by Inducing Vascular Permeability and Angiogenesis. Nat Commun (2018) 9:5395. doi: 10.1038/s41467-018-07810-w 30568162 PMC6300604

[126] Liu Q Rojas-Canales DM Divito SJ Shufesky WJ Stolz DB Erdos G. Donor Dendritic Cell-Derived Exosomes Promote Allograft-Targeting Immune Response. J Clin Invest (2016) 126:2805–20. doi: 10.1172/JCI84577 PMC4966303 27348586

[127] Marino J Babiker-Mohamed MH Crosby-Bertorini P Paster JT Leguern C Germana S. Donor Exosomes Rather Than Passenger Leukocytes Initiate Alloreactive T Cell Responses After Transplantation. Sci Immunol (2016) 1(1): aaf8759. doi: 10.1126/sciimmunol.aaf8759 27942611 PMC5142759

[128] Montecalvo A Shufesky WJ Stolz DB Sullivan MG Wang Z Divito SJ. Exosomes as a Short-Range Mechanism to Spread Alloantigen Between Dendritic Cells During T Cell Allorecognition. J Immunol (2008) 180:3081–90. doi: 10.4049/jimmunol.180.5.3081 18292531

[129] Sprent J Hurd M Schaefer M Heath W. Split Tolerance in Spleen Chimeras. J Immunol (1995) 154:1198–206. 7822792

[130] De Mestre A Noronha L Wagner B Antczak DF. Split Immunological Tolerance to Trophoblast. Int J Dev Biol (2010) 54:445–55. doi: 10.1387/ijdb.082795ad PMC2879498 19876828

[131] Gao Y Bado I Wang H Zhang W Rosen JM Zhang XH. Metastasis Organotropism: Redefining the Congenial Soil. Dev Cell (2019) 49:375–91. doi: 10.1016/j.devcel.2019.04.012 PMC6506189 31063756

[132] Gupta GP Massague J. Cancer Metastasis: Building a Framework. Cell (2006) 127:679–95. doi: 10.1016/j.cell.2006.11.001 17110329

[133] Valastyan S Weinberg RA. Tumor Metastasis: Molecular Insights and Evolving Paradigms. Cell (2011) 147:275–92. doi: 10.1016/j.cell.2011.09.024 PMC3261217 22000009

[134] Lopez-Soto A Gonzalez S Smyth MJ Galluzzi L. Control of Metastasis by NK Cells. Cancer Cell (2017) 32:135–54. doi: 10.1016/j.ccell.2017.06.009 28810142

[135] Zeeshan R Mutahir Z. Cancer Metastasis - Tricks of the Trade. Bosn J Basic Med Sci (2017) 17:172–82. doi: 10.17305/bjbms.2017.1908 PMC5581965 28278128

[136] Becker A Thakur BK Weiss JM Kim HS Peinado H Lyden D. Extracellular Vesicles in Cancer: Cell-To-Cell Mediators of Metastasis. Cancer Cell (2016) 30:836–48. doi: 10.1016/j.ccell.2016.10.009 PMC5157696 27960084

[137] Kong J Tian H Zhang F Zhang Z Li J Liu X. Extracellular Vesicles of Carcinoma-Associated Fibroblasts Creates a Pre-Metastatic Niche in the Lung Through Activating Fibroblasts. Mol Cancer (2019) 18:175. doi: 10.1186/s12943-019-1101-4 31796058 PMC6892147

[138] Grange C Brossa A Bussolati B. Extracellular Vesicles and Carried miRNAs in the Progression of Renal Cell Carcinoma. Int J Mol Sci (2019) 20(8): 1832. doi: 10.3390/ijms20081832 PMC6514717 31013896

[139] Guo Y Ji X Liu J Fan D Zhou Q Chen C. Effects of Exosomes on Pre-Metastatic Niche Formation in Tumors. Mol Cancer (2019) 18:39. doi: 10.1186/s12943-019-0995-1 30857545 PMC6413442

[140] Liu Y Cao X. Characteristics and Significance of the Pre-Metastatic Niche. Cancer Cell (2016) 30:668–81. doi: 10.1016/j.ccell.2016.09.011 27846389

[141] Li R Wen A Lin J. Pro-Inflammatory Cytokines in the Formation of the Pre-Metastatic Niche. Cancers (Basel) (2020) 12(12): 3752. doi: 10.3390/cancers12123752 PMC7764404 33322216

[142] Ji Q Zhou L Sui H Yang L Wu X Song Q. Primary Tumors Release ITGBL1-Rich Extracellular Vesicles to Promote Distal Metastatic Tumor Growth Through Fibroblast-Niche Formation. Nat Commun (2020) 11 1211. doi: 10.1038/s41467-020-14869-x 32139701 PMC7058049

[143] Donker RB Mouillet JF Chu T Hubel CA Stolz DB Morelli AE. The Expression Profile of C19MC microRNAs in Primary Human Trophoblast Cells and Exosomes. Mol Hum Reprod (2012) 18:417–24. doi: 10.1093/molehr/gas013 PMC3389496 22383544

[144] Morales-Prieto DM Ospina-Prieto S Schmidt A Chaiwangyen W Markert UR. Elsevier Trophoblast Research Award Lecture: Origin, Evolution and Future of Placenta miRNAs. Placenta (2014) 35 Suppl:S39–45. doi: 10.1016/j.placenta.2013.11.017 24378039

[145] Morales-Prieto DM Favaro RR Markert UR. Placental miRNAs in Feto-Maternal Communication Mediated by Extracellular Vesicles. Placenta (2020) 102:27–33. doi: 10.1016/j.placenta.2020.07.001 33218575

[146] Tong M Stanley JL Chen Q James JL Stone PR Chamley LW. Placental Nano-Vesicles Target to Specific Organs and Modulate Vascular Tone *In Vivo* . Hum Reprod (2017) 32:2188–98. doi: 10.1093/humrep/dex310 29040541

[147] Kumar A Deep G. Exosomes in Hypoxia-Induced Remodeling of the Tumor Microenvironment. Cancer Lett (2020) 488:1–8. doi: 10.1016/j.canlet.2020.05.018 32473240

[148] Truong G Guanzon D Kinhal V Elfeky O Lai A Longo S. Oxygen Tension Regulates the miRNA Profile and Bioactivity of Exosomes Released From Extravillous Trophoblast Cells - Liquid Biopsies for Monitoring Complications of Pregnancy. PLoS One (2017) 12:e0174514. doi: 10.1371/journal.pone.0174514 28350871 PMC5370130

[149] Rice GE Scholz-Romero K Sweeney E Peiris H Kobayashi M Duncombe G. The Effect of Glucose on the Release and Bioactivity of Exosomes From First Trimester Trophoblast Cells. J Clin Endocrinol Metab (2015) 100:E1280–1288. doi: 10.1210/jc.2015-2270 26241326

[150] Raimondo S Pucci M Alessandro R Fontana S. Extracellular Vesicles and Tumor-Immune Escape: Biological Functions and Clinical Perspectives. Int J Mol Sci (2020) 21(7):2286. doi: 10.3390/ijms21072286 PMC7177226 32225076

[151] Yin Z Fan J Xu J Wu F Li Y Zhou M. Immunoregulatory Roles of Extracellular Vesicles and Associated Therapeutic Applications in Lung Cancer. Front Immunol (2020) 11:2024. doi: 10.3389/fimmu.2020.02024 32983146 PMC7483575

[152] Abhange K Makler A Wen Y Ramnauth N Mao W Asghar W. Small Extracellular Vesicles in Cancer. Bioact Mater (2021) 6:3705–43. doi: 10.1016/j.bioactmat.2021.03.015 PMC8056276 33898874

[153] Stenqvist AC Nagaeva O Baranov V Mincheva-Nilsson L. Exosomes Secreted by Human Placenta Carry Functional Fas Ligand and TRAIL Molecules and Convey Apoptosis in Activated Immune Cells, Suggesting Exosome-Mediated Immune Privilege of the Fetus. J Immunol (2013) 191:5515–23. doi: 10.4049/jimmunol.1301885 24184557

[154] Atay S Gercel-Taylor C Suttles J Mor G Taylor DD. Trophoblast-Derived Exosomes Mediate Monocyte Recruitment and Differentiation. Am J Reprod Immunol (2011) 65:65–77. doi: 10.1111/j.1600-0897.2010.00880.x 20560914

[155] Atay S Gercel-Taylor C Taylor DD. Human Trophoblast-Derived Exosomal Fibronectin Induces Pro-Inflammatory IL-1β Production by Macrophages. Am J Reprod Immunol (2011) 66:259–69. doi: 10.1111/j.1600-0897.2011.00995.x 21410811

[156] Hendrix A Maynard D Pauwels P Braems G Denys H Van Den Broecke R. Effect of the Secretory Small GTPase Rab27B on Breast Cancer Growth, Invasion, and Metastasis. J Natl Cancer Inst (2010) 102:866–80. doi: 10.1093/jnci/djq153 PMC2886092 20484105

[157] Godakumara K Ord J Lättekivi F Dissanayake K Viil J Boggavarapu NR. Trophoblast Derived Extracellular Vesicles Specifically Alter the Transcriptome of Endometrial Cells and may Constitute a Critical Component of Embryo-Maternal Communication. Reprod Biol Endocrinol (2021) 19:115. doi: 10.1186/s12958-021-00801-5 34289864 PMC8293585

[158] Hoshino A Costa-Silva B Shen TL Rodrigues G Hashimoto A Tesic Mark M. Tumour Exosome Integrins Determine Organotropic Metastasis. Nature (2015) 527:329–35. doi: 10.1038/nature15756 PMC4788391 26524530

[159] Nguyen SL Ahn SH Greenberg JW Collaer BW Agnew DW Arora R. Integrins Mediate Placental Extracellular Vesicle Trafficking to Lung and Liver *In Vivo* . Sci Rep (2021) 11 4217. doi: 10.1038/s41598-021-82752-w 33602965 PMC7893009

